# Truncation of the Deubiquitinating Domain of CYLD in Myelomonocytic Cells Attenuates Inflammatory Responses

**DOI:** 10.1371/journal.pone.0016397

**Published:** 2011-01-20

**Authors:** Ageliki Tsagaratou, Dimitris L. Kontoyiannis, George Mosialos

**Affiliations:** 1 School of Biology, Aristotle University of Thessaloniki, Thessaloniki, Macedonia, Greece; 2 Institute of Immunology, Biomedical Sciences Research Center Al. Fleming, Vari, Greece; Karolinska Institutet, Sweden

## Abstract

The cylindromatosis tumor suppressor (CYLD) is a deubiquitinating enzyme that has been implicated in various aspects of adaptive and innate immune responses. Nevertheless, the role of CYLD in the function of specific types of immune cells remains elusive. In this report we have used conditional gene targeting in mice to address the role of the deubiquitinating activity of CYLD in the myelomonocytic lineage. Truncation of the deubiquitinating domain of CYLD specifically in myelomonocytic cells impaired the development of lethal LPS-induced endotoxic shock and the accumulation of thioglycollate-elicited peritoneal macrophages. Our data establish CYLD as a regulator of monocyte-macrophage activation in response to inflammatory stimuli and identify it as a potential target for therapeutic intervention in relevant inflammatory disorders in humans.

## Introduction

*Cyld* was identified as the predisposition gene for the tumor syndrome of familial cylindromatosis. It mediates the suppression of the NF-κB, JNK, p38 and Wnt pathways in a manner that depends on its deubiquitinating activity (reviewed in [1]). The use of mouse models of CYLD deficiency has implicated the protein in the regulation of multiple physiological processes including T cell development and activation, B cell homeostasis and function, osteoclastogenesis and spermatogenesis (Reviewed in [2]). Moreover, CYLD downregulation sensitizes mice to various pathological conditions that include chemically induced skin tumor formation as well as colitis-associated cancer [3]–[4]. Interestingly, in all these cases a broad range of CYLD-targeted proteins could be recognized, establishing CYLD as a multi-tasking deubiquitinating enzyme implicated in the fine tuning of many developmental and physiological processes.

A number of studies have provided compelling evidence for the implication of CYLD in various aspects of innate immunity. More specifically, it has been shown that CYLD constitutes a key negative regulator of NF-κB and JNK in macrophages from *Cyld* null mice that have been treated with CD40 or ligands against TLR4 and TLR2 [4]. Another study showed that *Cyld*-deficient mice were more resistant to lethal pulmonary infection caused by *Streptococcal pneumoniae*. This was attributed to enhanced p38 activation and subsequent elevated expression of PAI-1 [5], [6]. However, a third study did not recognize any difference in the activity of NF-κB or MAPKs in macrophages following treatment with TNF or TLR stimulation, using an independently derived mouse model of *Cyld* deficiency [7]. In all the aforementioned studies mice bearing obligatory null alleles were used making it impossible to discern the cell-specific contribution of CYLD to the observed phenotype [8].

In the present study we employed a conditional gene targeting approach to introduce a cell-specific deubiquitinating-domain-truncating mutation in the *Cyld* locus (*Cyld^Δ9^*) and investigate a plausible implication of CYLD in the development and function of the myelomonocytic lineage. In accordance with previous studies no obvious hematopoietic defects were observed. However, *Cyld^Δ9^* mice exhibited increased resistance to LPS induced endotoxic shock. Moreover, in an *in vivo* model of induced peritonitis CYLD-deficient macrophages failed to accumulate to the inflammatory site. Overall, our data demonstrate that *Cyld* is dispensable for hematopoiesis but unravel a cell intrinsic function of *Cyld* in monocyte-macrophage response to inflammatory stimuli.

## Results and Discussion

### Generation and characterization of *M-Cyld^Δ9^* mice

**Figure 1:**
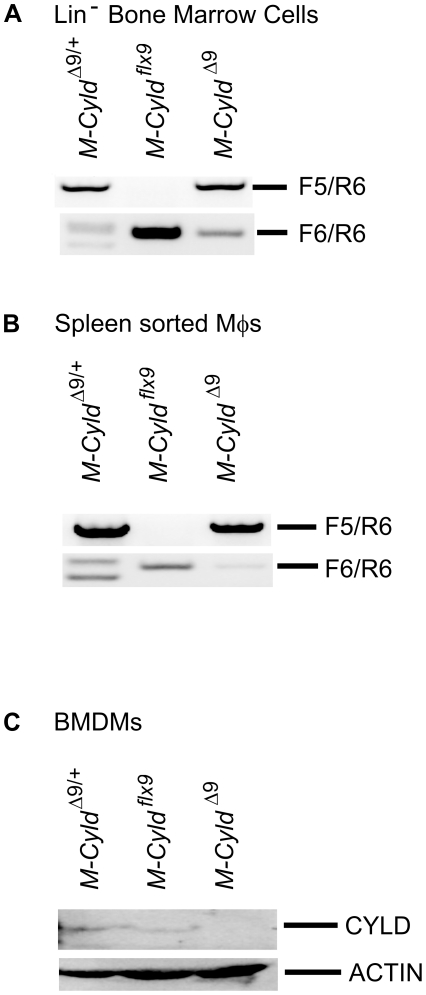
Generation and characterization of mice with inactivation of CYLD specifically in myelomonocytic cells. *Cyld* recombination is detected by genomic PCR in Lin^−^ precursor cells isolated from bone marrow (A) as well as in mature macrophages sorted from spleen (B). C) Immunoblotting of whole cell extracts from BMDMs isolated from control and *M-Cyld ^Δ9^* mice with anti-CYLD and anti-ACTIN antibodies.

**Figure 2:**
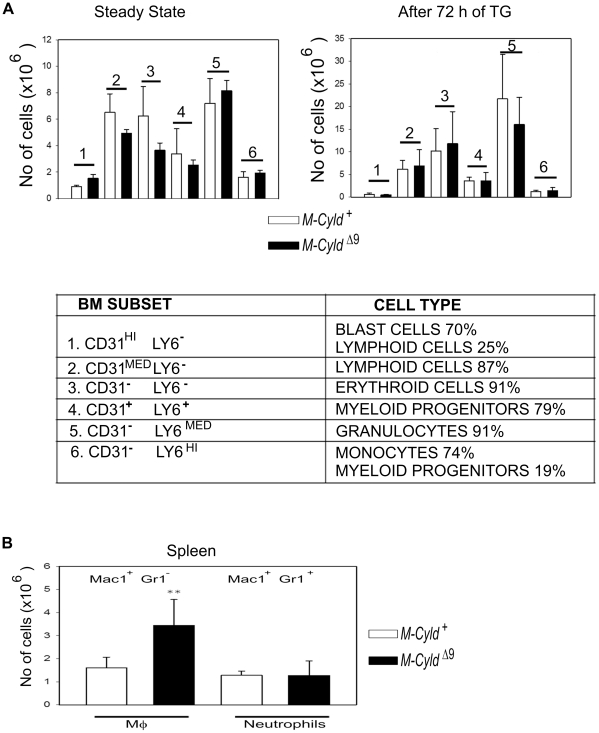
Normal hematopoietic development in *M-Cyld^Δ9^* mice. A) Enumeration of the indicated hematopoietic lineage cells (lower panel) by flow cytometric analysis of bone marrow cells for the expression of Ly6c and CD31 surface markers at steady state conditions (upper left panel) and after 72 h of thioglycollate treatment (upper right panel). In the first case 5 control and 3 *M-Cyld^Δ9^* mice were evaluated whereas in the second case 7 control and 5 *M-Cyld^Δ9^* mice were evaluated. B) Monocyte lineage populations in spleen. Enumeration of cells in the spleen that were collected from control (*M-Cyld^+^*) and mutant (*M-Cyld^Δ9^*) mice 72 hours after thioglycollate injection. Data are depicted as mean absolute numbers (±SEM) from n = 7 control and 5 *M-Cyld^Δ9^* mice at 8 weeks of age. The statistically significant difference in the macrophage populations (Mac1^+^ Gr1^−^) of control and mutant mice is depicted by two stars (p<0,001, Student's unpaired t test).

The generation of mice with loxP sites flanking the exon 9 of the *Cyld* locus (*Cyld ^flx9/flx9^*), has been described elsewhere [9]. By crossing these mice to *LysMcre* transgenic mice we aimed to render murine CYLD catalytically inactive in myelomonocytic cells, by truncating its catalytic domain through Cre-mediated excision of exon 9[9], [10]. In accordance with the expression of the Cre recombinase in myelomonocytic cells in *LysMCre-Cyld^flx9/flx9^* mice (termed *M-Cyld^Δ9^* mice from this point onwards), the effective excision of *Cyld* exon 9 was readily evident both in bone marrow purified progenitor cells (Fig. 1A) and in splenic sorted macrophages (Fig. 1B). Finally, immunoblotting with an anti-CYLD antibody indicated a nearly complete absence of full length CYLD protein in *M-Cyld^Δ9^* bone marrow derived macrophages (BMDMs, Fig. 1C). *M-Cyld^Δ9^* mice were viable and normal in appearance. Since CYLD has been implicated in T cell development [7], [11] the hematopoietic development in *M-Cyld^Δ9^* mice was evaluated. No gross hematopoietic defect was detected by flow cytometric analysis of the combined expression of Ly6C and CD31 surface markers both in steady state conditions and after thioglycollate-induced peritonitis that was adopted in order to evaluate the hematopoietic capacity of *M-Cyld^Δ9^* mice under stress (Fig. 2A). Interestingly, the higher numbers of splenic macrophages in *M-Cyld^Δ9^* mice (Figure 2B) further support the fact that there is not a hematopoietic defect.

### *M-Cyld^Δ9^* mice are less susceptible to LPS-induced endotoxic shock

**Figure 3:**
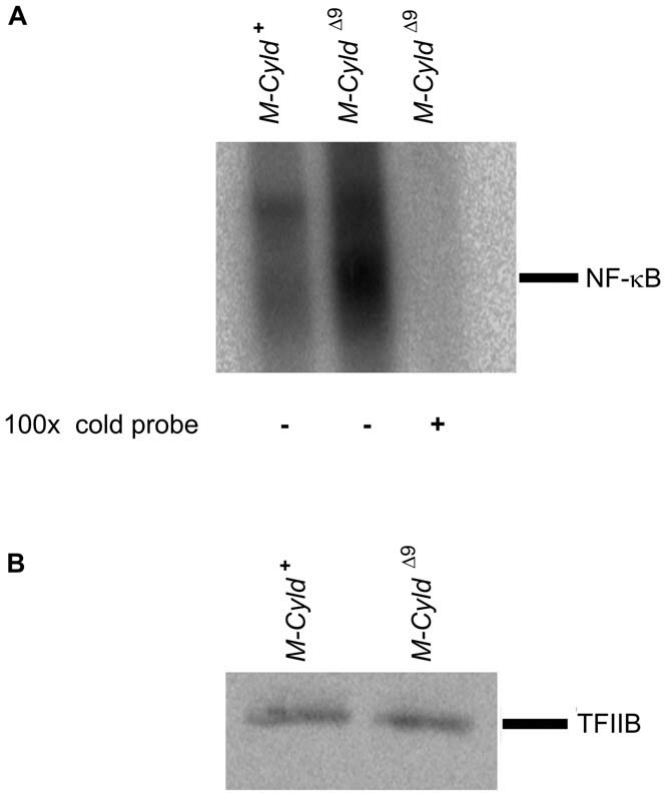
Elevated basal activity of NF-κB in *Cyld^Δ9^* BMDMs. A) EMSA of NF-κB DNA binding activity in BMDMs from control (*M-Cyld^+^*) and mutant (*M-Cyld^Δ9^*) mice in the absence or presence of 100-fold excess of unlabeled probe (100× cold probe) as indicated. The positions of NF-κB-containing complexes of the radiolabeled probe are shown. B) Immunoblot of nuclear extracts used in A with an anti-TFIIB antibody. Three mice per genotype were evaluated.

**Figure 4:**
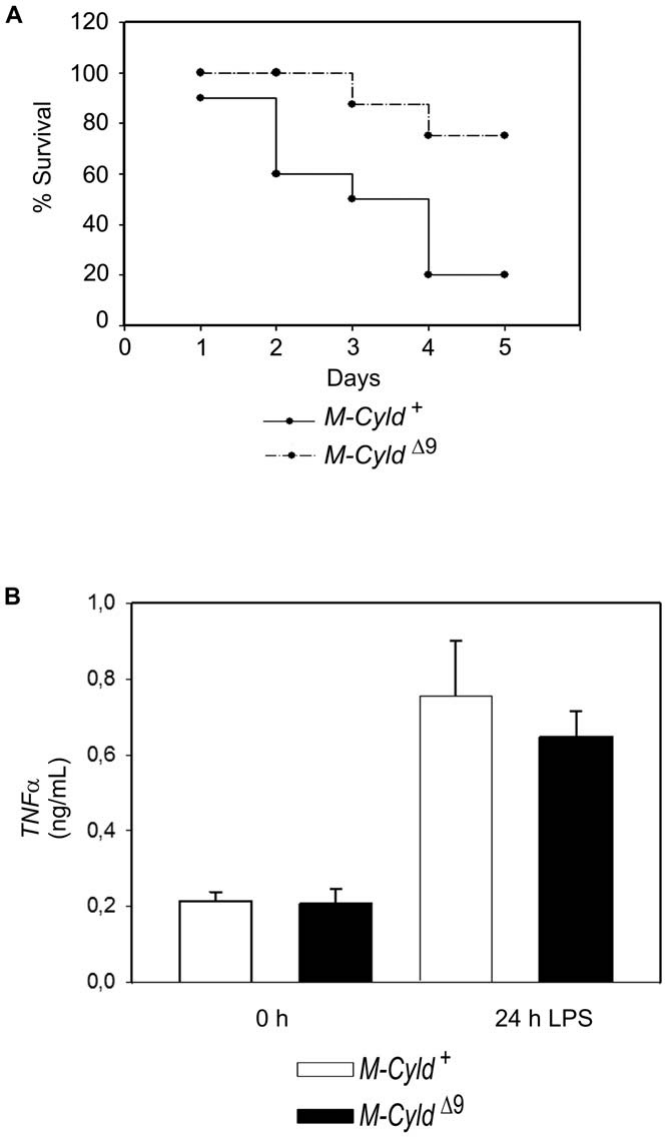
Attenuation of LPS-induced endotoxic shock in *M-Cyld^Δ9^* mice. A) Ten control (4 *Cyld ^flx9/+^* and 6 *Cyld ^flx9/+^ LysMcre*) and eight *M-Cyld^Δ9^* mice were subjected to LPS induced endotoxic shock (32 mg LPS/kg) and survival was monitored for the next 5 days. *M-Cyld^Δ9^* mice exhibited increased resistance to endotoxemia in comparison to control mice. Data are depicted as mean absolute numbers (±SEM) from n = 10 control and 8 *M-Cyld^Δ9^* mice at 8 weeks of age. (p value = 0,02 as assessed by Student's unpaired t test). B) Concentrations of TNFα were measured by enzyme-linked immunosorbent assay in supernatants from control and *M-Cyld^Δ9^* BMDMs before and 24 h after treatment with 100 ng/mL LPS. The results shown are the means (±S.D.) of triplicate measurements. Control BMDMs were isolated from 3 wild type and 4 *Cyld^flx9/+^LysMCre* mice and *M-Cyld^Δ9^* BMDMs were isolated from 4 mice.

Macrophages constitute a central player of the innate immune response. The NF-κB pathway has been implicated in many aspects of macrophage activation and function. *p50/NF-κB1* ^−/−^ mice show increased susceptibility to LPS-induced endotoxic shock due to decreased IL-10 production [12]. Interestingly, increased ubiquitination and degradation of p50 was observed in *Bcl3* ^−/−^ macrophages leading to the abolishment of LPS tolerance and the increased susceptibility of mice that lack BCL3 to LPS-induced shock [13]. Since CYLD has been shown to be a negative regulator of the NF-κB pathway in various cell types, its ability to modulate the activity of NF-κB in *Cyld^Δ9^* BMDMs was evaluated by EMSA. As expected, the basal NF-κB activity was significantly elevated in *Cyld^Δ9^* BMDMs in comparison to control BMDMs (Fig. 3). This finding prompted an investigation into the response of *M-Cyld^Δ9^* mice to LPS. *M-Cyld^Δ9^* and control mice were injected with 32 mg/kg of bacterial LPS. While only 20% of the control mice survived, the survival rate of *M-Cyld^Δ9^* mice reached 80%. Interestingly, *M-Cyld^Δ9^* mice not only showed increased resistance but also increased endurance since they died later than the control animals (Fig. 4A). Moreover, the levels of secreted TNFα following LPS treatment were not higher in *M-Cyld^Δ9^* BMDMs compared to control BMDMs. Instead, there was a tendency for lower secretion of TNFα by *M-Cyld^Δ9^* BMDMs following LPS treatment, even though this was not statistically significant (Figure 4B). The response of *M-Cyld^Δ9^* mice to LPS-induced endotoxemia was surprising, given the hyperactivation of NF-κB in *M-Cyld^Δ9^* BMDMs. A possible explanation for the observed phenotype could be the adoption of alternative activation by *M-Cyld^Δ9^* macrophages, which leads to the secretion of anti-inflammatory cytokines such as IL-10 [14]. Interestingly, NF-κB can exert an anti-inflammatory role besides its well known proinflammatory activity, which mediates the resolution of inflammation [15]. Moreover, it has been shown that conditional inactivation of IKK2 in myelomonocytic cells and subsequent endotoxin challenge of the resultant mice leads to increased endotoxin induced mortality and increased levels of IL-1β. Prolonged pharmacological inhibition of IKKβ, which interferes with NF-κB activation in the whole animal, also increased LPS-induced mortality and plasma IL-1β[16]. Therefore a plausible scenario would be that in *M-Cyld^Δ9^* mice the aberrant activation of NF-κB leads to suppression of IL1β generation as well as of the secretion of other inflammatory cytokines. Indeed, our findings are consistent with the resistance of *Cyld* null mice to lethality following acute lung injury by *Streptococcus pneumoniae* [6] and suggest that the lack of functional CYLD in macrophages may contribute to the increased survival of *Cyld* null mice. The availability of *M-Cyld^Δ9^* mice will permit the experimental evaluation of this hypothesis in order to pinpoint the cellular basis of *Cyld*-deficient mice resistance to lethal lung injury by *Streptococcus pneumonia*.

### *M-Cyld^Δ9^* mice show decreased accumulation of thioglycollate-elicited peritoneal macrophages

**Figure 5:**
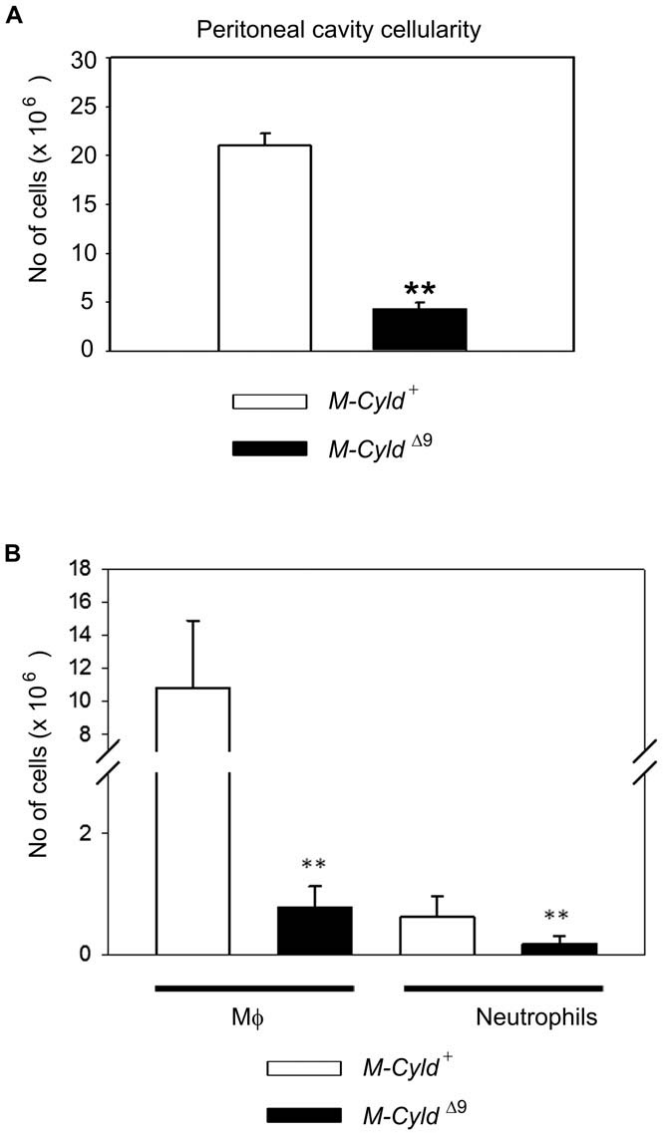
Impaired accumulation of thioglycollate-elicited peritoneal macrophages in *M-Cyld^Δ9^* mice. A) Enumeration of cells in the peritoneal cavity fluid that was collected from control (*M-Cyld^+^*) and mutant (*M-Cyld^Δ9^*) mice 72 hours after thioglycollate injection. Data are depicted as mean absolute numbers (±SEM) from n = 7 control and 7 *M-Cyld^Δ9^* mice at 8 weeks of age. The statistically significant difference in peritoneal cavity cellularity between control and mutant mice is depicted by two stars (p = 0,0000000064, Student's unpaired t test). (lower panel). B) Enumeration of macrophages (Mac1^+^Gr1^−^) and neutrophils (Mac1^+^Gr1^+^) from control (*M-Cyld^+^*) and mutant (*M-Cyld^Δ9^*) mice as assessed by flow cytometric analysis of cells harvested from the inflamed peritoneum 72 hours after thioglycollate injection by means of CD11b and Gr1 expression. Data are depicted as mean absolute numbers (±SEM) from n = 7 control and 7 *M-Cyld^Δ9^* mice at 8 weeks of age. The statistically significant difference in peritoneal cavity cellularity between control and mutant mice is depicted by two stars (p<0,01 unpaired t test).

In order to investigate further the implication of *Cyld* in the inflammatory response we employed an in vivo model of macrophage recruitment by inducing chemically aseptic peritonitis. Mice were intraperitoneally injected with thioglycollate, an extensively used inflammatory stimulus that induces the recruitment of neutrophils and macrophages to the peritoneal cavity [17]. In this model, neutrophil number reaches a maximum at 8 hours, while macrophages migrate with a slow time course, reaching a maximum at 72 hours and remaining at this level for at least 96 hours [18]. At 24 hours after thioglycollate injection similar numbers of cells were recovered from the peritoneal cavity of control and mutant mice (data not shown). However, at 72 hours after thioglycollate injection, there were significantly fewer cells recovered from the peritoneal cavity of *M-Cyld^Δ9^* mice compared with control mice (approximately 6 million from *M-Cyld^Δ9^* mice versus more than 20 millions from control mice, p = 0,0000000248) (Fig. 5A). Interestingly, the number of the recruited macrophages (CD11b^+^Gr1^−^) and neutrophils (CD11b^+^Gr1^+^) were dramatically decreased in *M-Cyld^Δ9^* mice in comparison to control mice (Fig. 5B). Notably, the *Cyld^Δ9^* macrophages that were recruited to the inflammatory site exhibited similar expression pattern of the activation maker F4/80 (data not shown). At this point it is not clear whether *Cyld^Δ9^* macrophages cannot be recruited due to defects in the migration process itself as a result of microtubule reorganization problems [19], [20] or if they cannot «perceive» the inflammatory stimulus due to signaling defects. Alternatively it is also possible that the reduced accumulation of *Cyld*-deficient macrophages at the site of inflammation reflects an increased propensity of these cells to death following their recruitment to the site of inflammation. Nevertheless, it is undisputable that in the context of chemically induced aseptic peritonitis *Cyld^Δ9^* macrophages are unable to respond properly.

The development and characterization of *M-Cyld^Δ9^* mice permitted the functional evaluation of CYLD in the myelomonocytic lineage and revealed an anti-inflammatory element of the functional profile of this molecule. Our findings add to a growing list of evidence that highlight the discrete and in certain cases contradictory tissue-specific activities of CYLD. For example, inactivation of CYLD in thymocytes leads to excessive apoptosis and impaired development [7], [11] whereas in peripheral T and B cells ablation of *Cyld* expression causes their hyperresponsiveness which can be associated with spontaneous autoimmune and inflammatory symptoms [21], [22]. The tissue-specific biological function of CYLD can be associated at least partly, with the differential role of the transcription factor NF-κB, which is regulated by CYLD, in different cellular contexts. Taken together with previous reports, the results of the present study highlight the importance of evaluating the functional role of genes by tissue-specific targeting, in order to clarify possibly contradictory findings that may stem from the compound effect of a gene's ablation in multiple tissues.

In summary, our experiments identified a cell-intrinsic requirement of functional CYLD for the efficient response of macrophages to different inflammatory stimuli and raise the possibility of targeting CYLD with inhibitory molecules as a pharmacological approach to control certain cases of pathogenic inflammation.

## Materials and Methods

### Ethics statement

Experiments on live animals were approved by the Hellenic Ministry of Rural Development-(Directorate of Veterinary Services, approval ID: 3926) and by Biomedical Sciences Research Center “Al. Fleming's Animal Research and Ethics Committee for compliance to FELASA regulations (approval ID: 2140).

### Mice

The generation of *Cyld ^flx9]/flx9^* mice has been described elsewhere [9]. *Cyld ^flx9]/flx9^* mice were crossed with *LysMcre* mice [10] in order to mutate *Cyld* in myelomonocytic cells. The mice were bred and maintained in the animal facilities of the Biomedical Sciences Research Centre “Alexander Fleming” under specific-pathogen free conditions.

### BMDM preparation

BMDM were generated as previously described [23].

### LPS induced endotoxic shock

Mice of 8 weeks age were intraperitoneally injected with a sublethal dose of 800 µg LPS (Sigma)/25 g animal weight and survival was monitored for 5 days.

### Electrophoretic mobility shift assay (EMSA)

Nuclear extracts were prepared by BMDMs and EMSA was performed as previously described [9].

The sequence of the oligonucleotides used for NF-κB with two tandemly repeated NF-κB binding sites (underlined) were as follows:

NF-κBf: 5′ -ATC AGG GAC TTT CCG CTG GGG ACT TT- 3′

NF-κBr: 5′-CGG AAA GTC CCC AGC GGA AAG TCC CT-3′

### *In Vivo* migration assay

Control and *M-Cyld ^Δ9^* mice were injected i.p. with 1 ml of 4% sterile thioglycollate (Becton Dickinson). 72 h later mice were sacrificed and the leukocyte number in the peritoneal lavage was assessed by a Coulter Counter. The percentage of the different cell types was assessed by Flow Cytometry.

### Flow Cytometry and Cell Sorting

Cell-associated fluorescence was analyzed by a FACS Aria or a FACSCantoII flow cytometer and the DIVA V6 software (Becton Dickinson). Sorting was performed with BD FACS Vantage SE II. Flow cytometry figures were prepared using the FlowJo software (Tree Star, Inc). Differences in populations were determined using the unpaired t test as calculated with Sigmaplot 9 statistical software.

### ELISA

For the TNFα (provided by W. Buurman, Nutrition and Toxicology Research Institute Maastricht, Maastricht, Netherlands) ELISA assay, BMDMs cultures (3×10^5^ cells/well) were incubated with 100 ng/ml LPS (*Salmonella enteriditis*; Sigma-Aldrich) and 24 h later the culture supernatants were collected.

### Antibodies

Cells were stained with Ly6c, CD31, CD11b, Gr1, CD16/32(BD Pharmingen), F4/80 (eBioscience), Lin- enriched fraction from bone marrow was prepared by staining with biotinylated Ter119, B220, Cd11b, Gr1, CD3 (eBioscience). Cylindromatosis and TFIIB were purchased from Santa Cruz. The anti-actin mouse monoclonal antibody was purchased from MP Biomedical Inc.
